# A descriptive study of the association of Carbapenem-resistant Enterobacterales with environmental justice of a Southern urban county, USA

**DOI:** 10.1371/journal.pone.0352253

**Published:** 2026-08-11

**Authors:** Alaina M. Beauchamp, Tanvi A. Ingle, Lauren N. Cooper, John J. Hanna, Marlon I. Diaz, Richard J. Medford

**Affiliations:** 1 School of Medicine, Department of Population Health, The University of Kansas Medical Center, Kansas City, Kansas, United States of America; 2 Peter O’Donnell Jr. School of Public Health, University of Texas Southwestern Medical Center, Dallas, Texas, United States of America; 3 UT Southwestern Medical School, University of Texas Southwestern Medical Center, Dallas, Texas, United States of America; 4 Clinical Informatics Center, University of Texas Southwestern Medical Center, Dallas, Texas, United States of America; 5 Information Services, ECU Health, Greenville, North Carolina, United States of America; 6 Division of Infectious Diseases and Geographic Medicine, Department of Internal Medicine, University of Texas Southwestern Medical Center, Dallas, Texas, United States of America; 7 Paul L. Foster School of Medicine, Texas Tech University Health Sciences Center, El Paso, Texas, United States of America; 8 Brody School of Medicine, Department of Internal Medicine, East Carolina University, Greenville, North Carolina, United States of America; Leibniz Institute DSMZ-German Collection of Microorganisms and Cell Cultures GmbH: Leibniz-Institut DSMZ-Deutsche Sammlung von Mikroorganismen und Zellkulturen GmbH, GERMANY

## Abstract

**Background:**

Carbapenem-resistant Enterobacterales (CRE) present a threat to global public health systems, yet the environmental contribution to antibiotic resistance prevalence has been understudied. The risk of antibiotic-resistant bacterium proliferating may be greater in neighborhoods experiencing environmental injustice (e.g., contaminated water, housing density). We examine spatial correlations between CRE detection rates and the Environmental Justice Index (EJI) across census tracts in an urban county.

**Methods:**

We analyzed data from electronic health records of two major health systems from 2015–2020. Using global spatial autocorrelation tests (i.e., Global Moran’s Index) and bivariate spatial lag regression models we analyzed the association of EJI components and local CRE rates.

**Findings:**

The overall EJI exhibited significant clustering in the study area (Moran’s I = 0.51) and a strong positive association with local CRE positive culture rates (β = 0.44, SE = 0.10). The health vulnerability domain of the EJI (e.g., chronic disease burden) demonstrated the strongest association with CRE rates (β = 0.75, SE = 0.13). Measures of built environment (β = 0.42, SE = 0.16), water pollution (β = 0.41, SE = 0.11), housing type (β = 0.34, SE = 0.13) and housing characteristics (β = 0.41, SE = 0.10) displayed significantly strong associations with local CRE positive culture rates.

**Interpretation:**

These findings highlight the complex interplay between environmental conditions, social determinants, and health outcomes, indicating that areas with poor environmental quality and higher social vulnerability exhibited elevated CRE positive rates. This emphasizes the potential relevance of environmental and social disparities to antibiotic-resistant organism burden such as CRE.

## Introduction

Antibiotic resistance represents one of the most pressing public health challenges of our time, with Carbapenem-resistant Enterobacterales (CRE) standing out as a particularly alarming example [1]. As a group of bacteria resistant to carbapenem antibiotics, CRE are often considered “superbugs” due to their high resistance to multiple antibiotics. This resistance not only complicates treatment strategies but also poses significant risks to public health systems globally.

The environmental dimension of antibiotic resistance, particularly regarding CRE, is complex and multifaceted. It involves the interplay of various factors across different geographies and neighborhoods. The spread of antibiotic resistance is not confined to clinical settings alone; it extends into the environment through multiple pathways [2]. For instance, the improper disposal of pharmaceutical waste, agricultural runoff containing antibiotics, and inadequate wastewater management can all contribute to the spread of resistant bacteria in the environment [3–5]. Investigation of a suburban creek near Chicago revealed high antimicrobial resistance in bacteria like Providencia alcalifaciens, demonstrating environmental reservoirs of antimicrobial resistance (AMR) in residential areas [6].

Socioeconomic factors and urbanization levels can also play a crucial role. In some neighborhoods, especially those with limited access to healthcare and poor sanitation, the risk of spread and exposure to antibiotic-resistant bacteria can be notably higher [7]. Environmental factors contributing to antibiotic resistance, particularly CRE, require a multidisciplinary approach. It spans microbiology, environmental science, epidemiology, and public health policy [8]. Understanding the environmental determinants of antibiotic resistance is crucial for developing effective strategies to combat its spread, not only within clinical settings but also in the wider community.

In summary, the topic of environmental hazards related to antibiotic resistance, particularly CRE, is a critical area of research that demands attention across various scientific disciplines. It is a global issue, and it impacts neighborhoods differently based on multiple environmental, social, and economic factors. Addressing this challenge requires a comprehensive understanding of potential ecological pathways associated with antibiotic resistance and implementation of targeted strategies to mitigate its impact on public health. The objective of this study is to provide a descriptive assessment of the relationship between environmental hazards and local CRE positive culture rates for an urban county in the Southern United States.

## Methods

### Study area and sample

Data from patient encounters were obtained from two prominent health systems: Texas Health Resources (THR) and the University of Texas Southwestern Medical Center (UTSW). These health systems are situated within the Dallas–Fort Worth metropolitan area in Texas. THR operates 29 acute care and critical access hospitals, as well as a network of urgent care centers and other outpatient facilities, mainly serving the Fort Worth region. UTSW is an academic health science center providing quaternary care, with two acute care hospitals and multiple outpatient clinics, primarily catering to the Dallas area. To contextualize the scale of these systems, in 2022, they collectively managed approximately 5.9 million visits, spanning emergency departments, outpatient services, and inpatient hospitalizations. Period-specific encounter volumes for 2015−2020 were not available, and the system capacity, along with patient volumes, may have varied over the study period, particularly due to disruptions associated with the COVID-19 pandemic. The frequency of cultures per year in the study period can be found in S1 Table. These health systems serve a large proportion of the population within Tarrant County, although patients residing in this county may choose to receive care at another hospital system.

THR and UTSW have comprehensive microbiology laboratories and utilize the same electronic health record system (Epic), which captured longitudinal data on demographics, microbiology, and laboratory results. The microbiology labs across these health systems process cultures using various standard techniques like broth microdilution, disk diffusion, and rapid molecular detection to process cultures and determine AMR. The microbiology labs performing the patient culture processing set the breakpoint standards, guided by the Clinical Laboratory Standards Institute (CLSI). Over the study period, the CLSI guidelines were revised [9], but were based on minimal inhibitory concentration results. Utilizing the electronic health data warehouses, we extracted all microbiologic culture results from various body sites (including urine, blood, respiratory, and body fluids such as ascites and pleural fluids, as well as PCR screening tests for methicillin-resistant Staphylococcus aureus [MRSA]; PCR screening tests for *Clostridioides difficile* were excluded) along with the associated phenotypic susceptibilities for adult patients (aged ≥18 years) who had inpatient or outpatient encounters at either institution between January 1, 2015, and December 31, 2020. To prevent double-counting the same infection, any cultures for the same individual during the same encounter with the same organism and resistance mechanism were considered one case. We counted only the first occurrence if subsequent cultures were obtained within a period of ninety days from the initial positive culture. Clinical classification of cultures was not systematically available in the data; thus no distinction was made between “true infections” and colonization among the collected cultures. For the patient’s index culture, antibiotic susceptibility was determined using free-text notations associated with the microbiological culture results. Any culture with Enterobacterales that was labeled as “non-susceptible,” “resistant,” or “intermediate” to a carbapenem as documented in the laboratory testing results was labeled as CRE. Organisms included as CRE can be found in the S2 Table. The definition of a CRE positive culture result was consistently applied to all years of study.

The residential address reported in the patient encounter with a sampled culture was geocoded using the US Census Bureau’s Census Geocoder Application Programming Interface to identify the census tract of the patient residence. Records with addresses that could not be geocoded were excluded, representing < 10% of total cultures.

### Patient consent

This research (STU-2023–0583) was approved by the UTSW Institutional Review Board. As secondary retrospective data use research, this study received a waiver of consent due to the deidentified nature of the data used in the study. Data was extracted beginning August 1, 2023. Additional details on the methodology used in this study can be found in Cooper et al., 2024 [10].

### Measures

Patient-level CRE culture results were aggregated to the patient’s residential census tract-level across 2015–2020. We measured the spatial distribution of our outcome variable Carbapenem-resistant Enterobacterales (CRE) positive culture rate per 1,000 population in each census tract in the study area. Population estimates used as denominators in the CRE positive culture rate calculation used the 2020 Decennial Census population estimates. CRE positive culture rates were adjusted using Empirical Bayes standardization which stabilizes estimates by shrinking rates in low-count tracts toward the overall mean, reducing the influence of random variation.

The Environmental Justice Index (EJI) was developed by the Centers for Disease Control and Prevention (CDC) in 2022 to serve as a tool for identifying and addressing disparities in environmental health impacts across different communities. Environmental justice is a concept that ensures that all communities, especially the disadvantaged, are protected from disproportionate environmental harms and risks.

The EJI is composed of multiple indicators that represent various dimensions of environmental justice, including exposure to pollutants, health vulnerabilities, and socio-economic factors. The primary data sources for these indicators include national databases such as the U.S. Census, the Environmental Protection Agency data on air and water quality, and health data from the CDC. Each indicator is carefully selected based on its relevance to public health and ability to reflect disparities among different population groups.

Each component of the EJI is normalized to ensure comparability across different metrics. The normalization process involves converting each indicator into a common scale, typically ranging from 0 (least disadvantageous) to 100 (most disadvantageous). These normalized scores are then aggregated using a weighted average method, where weights reflect the relative importance of each indicator to the overall environmental justice assessment.

### Statistical analysis

We conducted an exploratory descriptive ecological analysis of the spatial distribution of CRE positive cultures per 1,000 population in a census tract. We tested spatial autocorrelation (i.e., clustering) of community indicators using the Global Moran’s Index (p-value tested with 499 permutations). To quantify the spatial co-clustering of census tract CRE positive culture rates and EJI components, we computed the global bivariate Moran’s I statistic, defined as the mean of the local bivariate LISA (Local Indicators of Spatial Association) statistics following the method of Anselin, Syabri, and Smirnov (2002) [11]. This statistic measures the correlation between CRE positive culture rates in a given tract and EJI values in neighboring tracts, with significance again assessed via 499 permutation inference. We then estimated bivariate (unadjusted) spatial lag regression models to assess the association between each EJI indicator and tract-level CRE positive culture rates. This exploratory approach was chosen to characterize the breadth of environmental justice dimensions that spatially co-distribute with CRE burden, rather than to identify independent predictors. Spatial dependence was modeled using a row-standardized queen contiguity spatial weights matrix. As a sensitivity analysis, we re-estimated all domain- and module-level models using a rook contiguity spatial weights matrix. These results are presented in S3 Table (Supplementary Material).

All initial data processing was completed with the Python (v3.10.5) programming language. Maps were created in ArcGIS Pro (v3.2.1). All spatial analyses were completed using GeoDa software (v1.20.0.22).

## Results

A total of 448 census tracts in the county were included in the analysis. Across the study period (2015–2020), 1,615 CRE-positive cultures were identified. The median tract-level CRE count was three (interquartile range [IQR]: 1–6), with 58 tracts (13%) recording zero CRE detection events. Tract-level population denominators of the study area had a median of 4,541 adult residents (IQR: 3,513–5,728).

**Fig 1:**
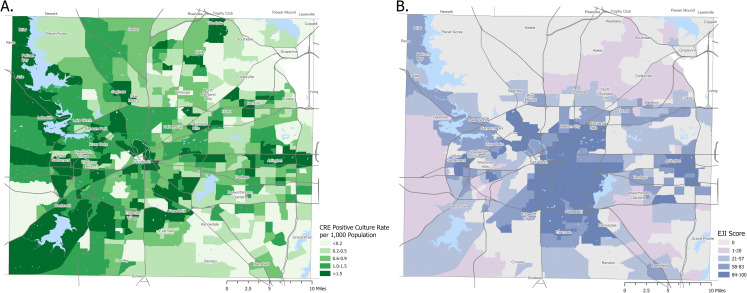
Spatial distribution of quantiles for CRE positive cultures per 1,000 population (Panel A) and Environmental Justice Index (Panel B) across census tracts in the study area. Basemap data from the U.S. Census Bureau TIGER/Line Shapefiles: https://www.census.gov/geographies/mapping-files/time-series/geo/tiger-line-file.html.

We observed significant spatial autocorrelation across multiple environmental, social, and health variables, as measured by Global Moran’s I values, indicating non-random spatial patterns of CRE positive cultures. Overall, patterns of EJI clustering were consistent with the clustering of CRE positive cultures to a small degree with a bivariate local Moran’s Index value of 0.21 (p < 0.002), as seen in Table 1. Moran’s I values were significant across all major categories, emphasizing spatial clustering of CRE positive culture rates and their association with environmental and social determinants. Notably, the overall EJI showed a high spatial autocorrelation (Moran’s I = 0.51, p < 0.05) and a strong positive association with local CRE positive culture rates (β = 0.44, standard error [SE] = 0.10). Fig 1 displays the spatial distribution of CRE positive cultures per 1,000 population (Panel A) and the EJI ranks (Panel B) across census tracts in the study area.

**Table 1:** Spatial autocorrelation and association of Carbapenem-resistant *Enterobacterales* positive culture rate with the Environmental Justice Index across census tracts in a Southern Urban county (2015-2020).

		CRE
	*Moran’s I*	*Β (SE)*
*EJI Overall*	0.51*	0.44 (0.10)**
*Environmental Burden*	0.35*	0.26 (0.11)*
Air Pollution	0.35*	0.31 (0.10)*
Ozone	0.36*	0.24 (0.08)*
PM2·5	0.35*	0.25 (0.12)*
Diesel Particulate Matter	0.36*	0.26 (0.11)*
Air Toxics Cancer Risk	0.34*	0.25 (0.11)*
Potentially Hazardous & Toxic Sites	0.39*	0.11 (0.11)
National Priority List Sites	0.40*	0.06 (0.23)
Toxic Release Inventory Sites	0.38*	0.10 (0.10)
Treatment, Storage, and Disposal Sites	0.44*	0.42 (0.19)*
Risk Management Plan Sites	0.36*	−0.01 (0.10)
Coal Mines	0.42*	0.33 (0.32)
Built Environment	0.36*	0.42 (0.16)*
Recreational Parks	0.27*	0.34 (0.16)*
Houses Built Pre-1980	0.54*	0.42 (0.11)*
Walkability	0.32*	0.16 (0.13)
Transportation Infrastructure	0.31*	0.29 (0.11)*
High-Volume Roads	0.42*	0.37 (0.11)*
Railways	0.49*	0.16 (0.11)
Airports	0.25*	0.10 (0.13)
Water Pollution	0.48*	0.41 (0.11)*
Impaired Surface Water	0.48*	0.41 (0.11)*
*Social Vulnerability*	0.48*	0.36 (0.10)*
Racial/Ethnic Minority Status	0.40*	0.23 (0.10)*
Socioeconomic Status	0.47*	0.32 (0.10)*
Poverty	0.49*	0.32 (0.10)*
No High School Diploma	0.49*	0.27 (0.10)*
Unemployment	0.28*	0.26 (0.12)*
Housing Tenure	0.27*	0.26 (0.11)*
Housing Burdened Lower-Income Households	0.35*	0.28 (0.11)*
Lack of Health Insurance	0.40*	0.28 (0.09)*
Lack of Broadband Access	0.57*	0.39 (0.12)*
Household Characteristics	0.40*	0.41 (0.10)**
Age 65 and Older	0.33*	0.69 (0.13)**
Age 17 and Younger	0.35*	0.05 (0.09)
Civilian with Disability	0.32*	0.80 (0.13)**
Speaks English “Less than Well”	0.37*	0.15 (0.09)
Housing Type	0.20*	0.34 (0.13)*
Group Quarters	0.16*	0.31 (0.12)*
Mobile Homes	0.16*	0.23 (0.13)
*Health Vulnerability*	0.55*	0.75 (0.13)**
Pre-Existing Chronic Disease Burden		
Asthma	0.52*	0.16 (0.11)
Cancer	0.31*	0.49 (0.11)**
High Blood Pressure	0.41*	0.60 (0.08)**
Diabetes	0.51*	0.44 (0.08)**
Poor Mental Health	0.44*	0.17 (0.08)*

*p < 0.05; **p < 0.0001.

The Lead Mines measure of the Potentially Hazardous & Toxic Sites subdomain, which is a component of the Environmental Burden domain, was removed from the table given the lack of sites in the study area.

Concerning the environmental burden category of the EJI, we noted that air pollution, including subcategories such as ozone (Moran’s I = 0.36, p < 0.05; β = 0.24, SE = 0.08), PM2.5 (Moran’s I = 0.35, p < 0·05; β = 0.25, SE = 0.12), and diesel particulate matter (Moran’s I = 0.36, p < 0.05; β = 0.26, SE = 0.11), all demonstrated significant clustering and associations (i.e., regression coefficients), indicating spatial co-occurrence of higher pollution levels and increased rates of CRE positive cultures.

In our analysis, the built environment, particularly older housing (houses built pre-1980), also showed a high Moran’s I value (I = 0.54, p < 0.05) and a significant positive regression coefficient (β = 0.42, SE = 0.11), indicating that older infrastructure was associated with higher CRE positive culture rates. Additionally, water pollution, indicated by impaired surface water (i.e., elevated levels of waterborne pathogens or contamination by toxic substances), had significant Moran’s I values (I = 0.48, p < 0.05) and regression coefficients (β = 0.41, SE = 0.11), consistent with an association between environmental conditions and health outcomes.

Social vulnerability factors of the EJI such as socioeconomic status (Moran’s I = 0.47, p < 0.05; β = 0.32, SE = 0.10), racial/ethnic minority status (Moran’s I = 0.40, p < 0.05; β = 0.23, SE = 0.10), and lack of broadband access (Moran’s I = 0.57, p < 0.05; β = 0.39, SE = 0.12) were notably correlated with CRE positive culture rates, highlighting the spatial correlation between socioeconomic disparities and CRE detection.

The health vulnerability category of the EJI also showed strong spatial autocorrelation (Moran’s I = 0.55, p < 0.05) and was significantly associated with the prevalence of CRE positive cultures, particularly in populations with pre-existing conditions such as high blood pressure (Moran’s I = 0.41, p < 0.05; β = 0.60, SE = 0.08) and diabetes (Moran’s I = 0.51, p < 0.05; β = 0.44, SE = 0.08).

**Fig 2:**
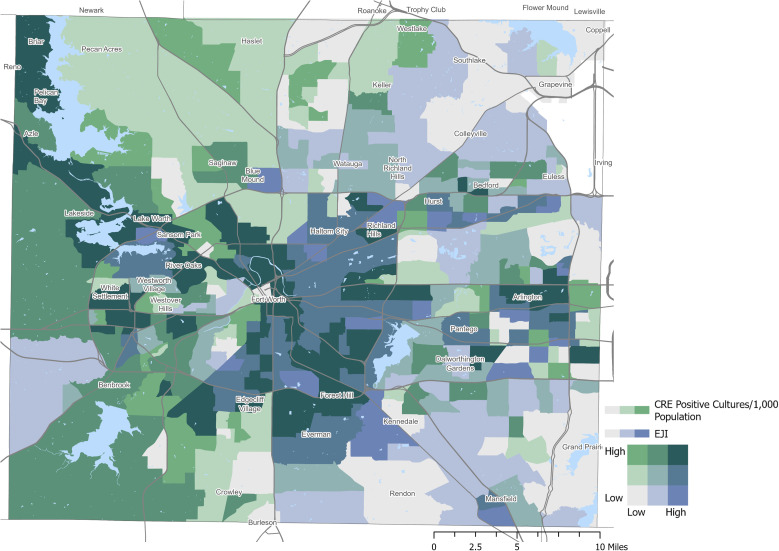
Overlap of high/low tertiles of CRE positive cultures per 1,000 population and overall Environmental Justice Index across census tracts in the study area. Basemap data from the U.S. Census Bureau TIGER/Line Shapefiles: https://www.census.gov/geographies/mapping-files/time-series/geo/tiger-line-file.html.

Fig 2 displays the overlap of areas across high/low tertiles of CRE positive culture rates and the overall EJI. Areas in dark green/blue are classified as having both a high CRE positive culture rate and a high EJI score, indicating localities that have prevalent environmental hazards and antimicrobial resistance concerns. Those areas in blue have more environmental justice concerns, but lower rates of CRE; and areas in green have less environmental justice concerns, and higher rates of CRE.

## Discussion/Conclusions

These results underline the complex interplay between environmental conditions, social determinants, and health outcomes, suggesting that areas characterized by poor environmental quality and higher social vulnerability exhibited higher CRE positive culture rates. The study highlights the potential relevance of environmental and social inequalities to the burden of antibiotic-resistant organisms such as CRE.

Air pollution indicators in this study (e.g., ozone, PM2.5, diesel particulate matter) demonstrated significant spatial associations with tract-level CRE rates. These findings are consistent with a growing body of literature linking air pollutants exposure to antibiotic-resistance patterns [12–17]. The spread of PM2.5 and diesel particulate matter from car exhaust, has been found to drive antibiotic resistance for organisms including CRE [18]. Several mechanisms have been proposed to explain this relationship, including pollutant particles serving as surfaces for bacterial exchange of resistance genes and facilitating airborne transmission of resistant organisms [12,13,16,17]. Our study design does not allow us to distinguish whether air pollution directly promotes CRE proliferation or whether these spatial associations reflect shared upstream determinants such as neighborhood deprivation. Incorporating environmental sampling alongside clinical data would help clarify these pathways.

Water pollution, measured by impaired surface water, was significantly associated with local CRE rates. Previous studies have identified bodies of water as potential reservoirs for CRE, particularly where agricultural runoff and hospital effluents enter waterways. While local data on the presence of CRE in our study area water supply is not available, the spatial co-occurrence of impaired water quality and elevated CRE rates is consistent with these environmental reservoir hypotheses. This association warrants further investigation with direct environmental sampling.

Socioeconomic vulnerability indicators, including poverty, lack of health insurance, and lack of broadband access were spatially associated with CRE rates. These findings are consistent with prior literature linking lower socioeconomic status to higher burdens of AMR [7,19]. Proposed mechanisms include limited access to health care leading to delayed or inappropriately treated CRE infections [19], higher household density facilitating transmission, and proximity to local long-term care facilities (e.g., nursing facilities) which can be reservoirs for these pathogens [20–23].

Built environment indicators, particularly pre-1980 housing, showed significant associations with CRE rates. Older housing stock may reflect neighborhood disinvestment and infrastructure conditions that could facilitate pathogen transmission, though the specific mechanisms linking housing age to CRE burden are not well-established [24]. The association between food desert prevalence and microbiome diversity [25] represents another plausible but untested pathway, with 14% of the residents of our study area living in a food desert.

The health vulnerability domain of the EJI showed the strongest associations with CRE rates, particularly for high blood pressure, diabetes, and cancer prevalence. This likely reflects, at least in part, the increased contact with healthcare facilities and immunosuppression associated with chronic disease [26,27], which are well-established clinical risk factors for CRE infections and associated morbidity and mortality [28]. However, because these indicators are themselves spatially correlated with socioeconomic and environmental vulnerability, disentangling the independent contribution of chronic disease burden from other EJI components was not feasible for this ecological analysis.

This study has several limitations that should be considered when interpreting the results. First, the ecological nature of the study design limits our ability to establish causality between environmental justice indicators and CRE positive culture rates. Our findings are based on aggregated data at the census tract level, which may obscure individual-level factors that contribute to CRE detection. In addition, CRE rates were derived from clinical encounters at two health systems, and residents of the study area county may seek care elsewhere. Spatial patterns may therefore partly reflect differential healthcare access, catchment area coverage, and testing intensity rather than true community burden. Next, as an ecological study, associations observed at the census tract level may not hold at the individual level. Additionally, due to the reliance on existing databases, we could not differentiate between true CRE infections and colonization, which may overestimate or misclassify the burden of CRE in the study population. Another limitation is the lack of data on environmental CRE reservoirs in the study area, such as contamination of local water sources or soil, which could provide further insight into environmental transmission pathways. This study also used EJI indicators, which are highly correlated with one another, and our exploratory bivariate association models cannot provide a determination of which specific dimensions of environmental injustice independently drive CRE burden. Finally, given that we used aggregated data across 2015–2020, we were unable to assess the temporal trends or changes in CRE epidemiology over the study period. Despite these limitations, this study provides important insights into the spatial relationship between environmental justice and antimicrobial resistance, particularly CRE, and underscores the need for further research in this area.

This study highlighted community disparities associated with the local rate of CRE positive cultures, suggesting an overlap of concerns for environmental justice and antimicrobial resistance. We described this association to highlight areas that may fall into the upstream risk factors for local antimicrobial resistance, in this case for CRE. While this study cannot show a direct relationship between these factors and the incidence of CRE detection, it underscores the potential relevance of social determinants of health and environmental conditions to AMR patterns, and supports further investigation into whether addressing these upstream factors could contribute to CRE mitigation strategies.

## Research in context

### Evidence before this study

Despite growing awareness of environmental contributions to antimicrobial resistance, research directly linking environmental justice indicators to carbapenem-resistant Enterobacterales (CRE) detection remains scarce. Most studies have focused on clinical risk factors or isolated environmental exposures (e.g., wastewater, air pollution) without assessing their cumulative impact within community contexts. To date, no comprehensive reviews or large-scale studies have systematically examined the spatial relationship between environmental injustice and CRE burden across neighborhoods, particularly in the United States.

### Added value of this study

This is one of the first studies to spatially link census tract-level CRE positive culture rates with components of the CDC’s Environmental Justice Index in a major Southern urban county. By integrating electronic health records with environmental and sociodemographic indicators, we identified strong spatial clustering of CRE positive cultures in areas with poor air and water quality, aging infrastructure, and heightened social vulnerability. The study provides a novel geospatial framework to understand upstream environmental and social correlates of antimicrobial resistance.

### Implications of all the available evidence

Taken together, existing and emerging evidence suggests that environmental justice may be an underrecognized dimension relevant to antimicrobial resistance patterns. Our findings suggest that future surveillance and research strategies should consider environmental and social determinants when characterizing antimicrobial resistance patterns in communities. Integrating environmental equity into antimicrobial resistance mitigation efforts may reduce local infection burdens and improve health outcomes in disadvantaged populations.

## Supporting information

S1 Table Count of Carbapenem resistant Enterobacterales cultures across each year in the study period. (DOCX)

S2 Table Enterobacterales organisms included. (DOCX)

S3 Table Sensitivity analysis of alternative spatial weight specifications. (DOCX)
