# Supplementary material for: A descriptive study of the association of Carbapenem-resistant Enterobacterales with environmental justice of a Southern urban county, USA
Source: PLoS One. 2026 Aug 11;21(8):e0352253. doi: 10.1371/journal.pone.0352253 (PMC13460609; doi:10.1371/journal.pone.0352253)
Supplement: Supplementary file 2 [file pone.0352253.s002.docx]

**Table S2.**

**Organisms**

CITROBACTER AMALONATICUS

CITROBACTER BRAAKII

CITROBACTER FREUNDII

CITROBACTER YOUNGAE

ENTEROBACTER AEROGENES

ENTEROBACTER AMNIGENUS 2

ENTEROBACTER ASBURIAE

ENTEROBACTER CLOACAE

ENTEROBACTER CLOACAE COMPLEX

ENTEROBACTER GERGOVIAE

ENTEROBACTER HORMAECHEI

ENTEROBACTER KOBEI

ESCHERICHIA COLI

KLEBSIELLA OXYTOCA

KLEBSIELLA PNEUMONIAE

KLEBSIELLA PNEUMONIAE SSP OZAENAE

KLEBSIELLA PNEUMONIAE SSP PNEUMONIAE

PROTEUS MIRABILIS

PROTEUS MIRABILIS/PENNERI

PROTEUS PENNERI

PROTEUS SPECIES

PROTEUS VULGARIS

PROTEUS VULGARIS/PENNERI

PROVIDENCIA RETTGERI

PROVIDENCIA STUARTII

SERRATIA FONTICOLA

SERRATIA LIQUEFACIENS

SERRATIA MARCESCENS
