# Supplementary material for: A descriptive study of the association of Carbapenem-resistant Enterobacterales with environmental justice of a Southern urban county, USA
Source: PLoS One. 2026 Aug 11;21(8):e0352253. doi: 10.1371/journal.pone.0352253 (PMC13460609; doi:10.1371/journal.pone.0352253)
Supplement: Supplementary file 3 [file pone.0352253.s003.docx]

**Table S3.**

|  | **CRE** | |
| --- | --- | --- |
|  | Model 1 | Model 2 |
|  | ***Β (SE)*** | ***Β (SE)*** |
| *EJI Overall* | 0·44 (0·10)** | 0.46 (0.10)** |
| *Environmental Burden* | 0·26 (0·11)* | 0.28 (0.11)* |
| Air Pollution | 0·31 (0·10)* | 0.23 (0.10)* |
| Potentially Hazardous & Toxic Sites | 0·11 (0·11) | 0.13 (0.11) |
| Built Environment | 0·42 (0·16)* | 0.45 (0.16)* |
| Transportation Infrastructure | 0·29 (0·11)* | 0.30 (0.11)* |
| Water Pollution | 0·41 (0·11)* | 0.42 (0.11)* |
| *Social Vulnerability* | 0·36 (0·10)* | 0.39 (0.10)* |
| Racial/Ethnic Minority Status | 0·23 (0·10)* | 0.24 (0.10)* |
| Socioeconomic Status | 0·32 (0·10)* | 0.34 (0.10)* |
| Household Characteristics | 0·41 (0·10)** | 0.43 (0.10)* |
| Housing Type | 0.34 (0·13)* | 0.37 (0.13)* |
| *Health Vulnerability* | 0·75 (0·13)** | 0.78 (0.13)** |
| Footnote: Model 1 = unadjusted spatial lag regression model weighted using queens contiguity spatial weights matrix; Model 2 = unadjusted spatial lag regression model weighted using rook contiguity spatial weights matrix | | |
