# Supplementary material for: A descriptive study of the association of Carbapenem-resistant Enterobacterales with environmental justice of a Southern urban county, USA
Source: PLoS One. 2026 Aug 11;21(8):e0352253. doi: 10.1371/journal.pone.0352253 (PMC13460609; doi:10.1371/journal.pone.0352253)
Supplement: Supplementary file 4 [file pone.0352253.s001.docx]

**Table S1.**

| Year | 2015 | 2016 | 2017 | 2018 | 2019 | 2020 |
| --- | --- | --- | --- | --- | --- | --- |
| Cultures, *n* | 55 | 44 | 557 | 560 | 189 | 214 |
